# Supplementary material for: Comparison and optimization of cellular neighbor preference methods for quantitative tissue analysis
Source: Nat Commun. 2026 Apr 15;17:3514. doi: 10.1038/s41467-026-71699-z (PMC13083810; doi:10.1038/s41467-026-71699-z)
Supplement: Supplementary file 1 — Supplementary Information [file 41467_2026_71699_MOESM1_ESM.pdf]

S1 Supplementary information

Comparison and Optimization of Cellular Neighbor Preference Methods for Quantitative Tissue Analysis

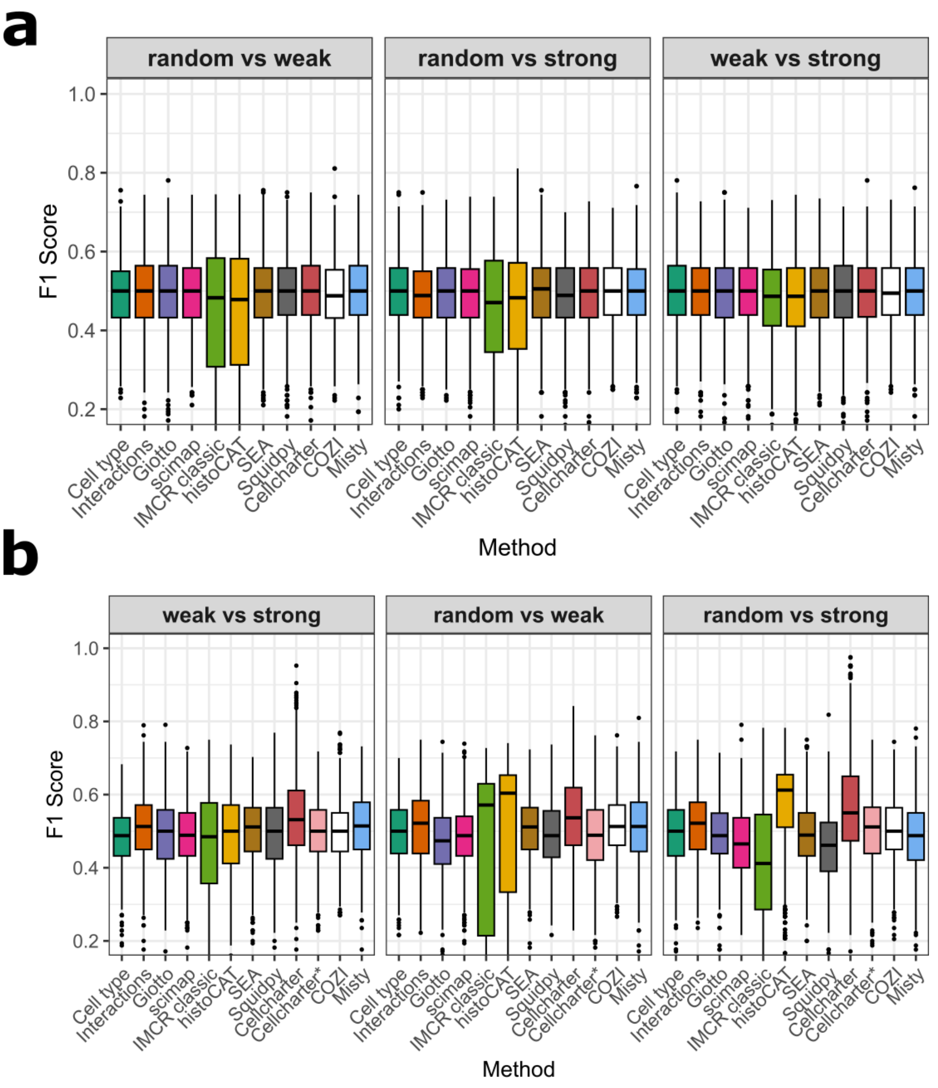

Supplementary Fig. 1: Baseline F1 scores after random label shuffling of NEP results. (a) Self-preference dataset (cell type red). Mean baseline F1-scores for cohort distinction tasks across red cell type abundance groups are shown as boxplots. Cohort comparisons include *weak vs. strong*, *random vs. weak*, and *random vs. strong*. Labels were randomly shuffled to establish the baseline. (b) Cross-preference dataset (red to yellow). Baseline mean F1-scores for the same cohort distinction tasks across yellow cell abundance groups are shown as boxplots. n = 100 samples per cohort were simulated and analyzed. Data are presented as box plots showing the median (centre line), interquartile range (box; 25th–75th percentiles), and whiskers extending to 1.5 × IQR. Outliers are shown as individual points. Source data are provided as a Source Data file.

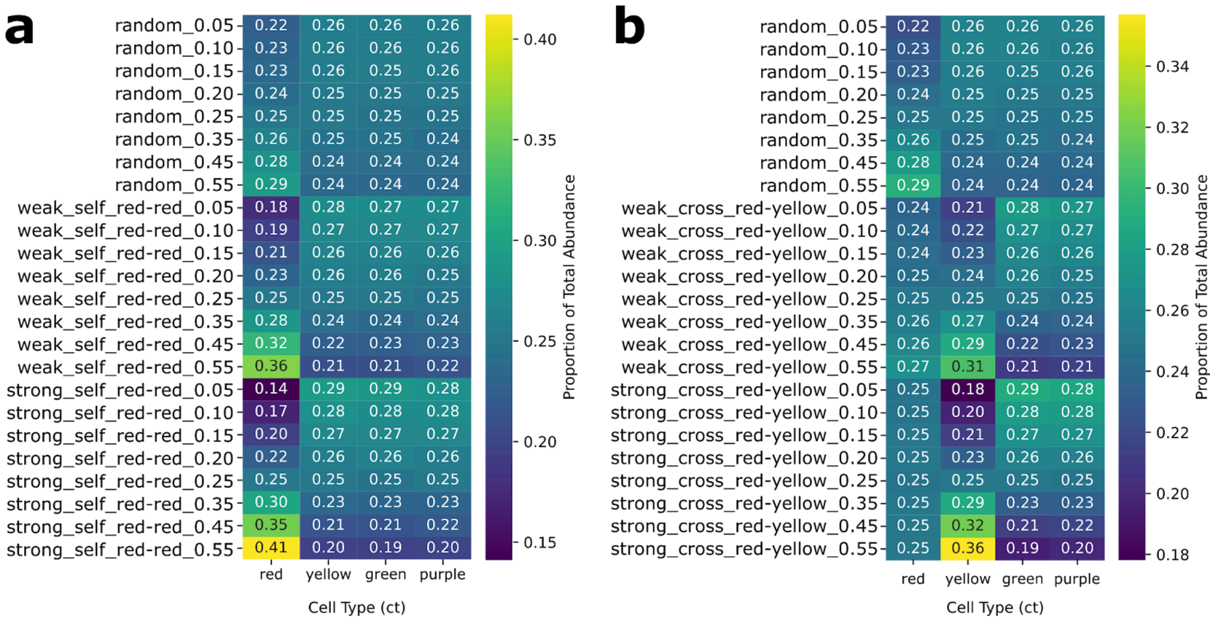

Supplementary Fig. 2: Differences between simulated and target cell type abundances in IST cohorts. (a) Symmetric dataset with self-preference of the red cell type. (b) Asymmetric dataset with cross-preference of the red cell type toward the yellow cell type. For each cohort, the mean proportions (n = 100 simulations) of all four simulated cell types are shown. Y-axis labels indicate the cohort name, followed by the parameterized red cell type abundance (ranging from 0.05–0.55; see Methods). Source data are provided as a Source Data file.

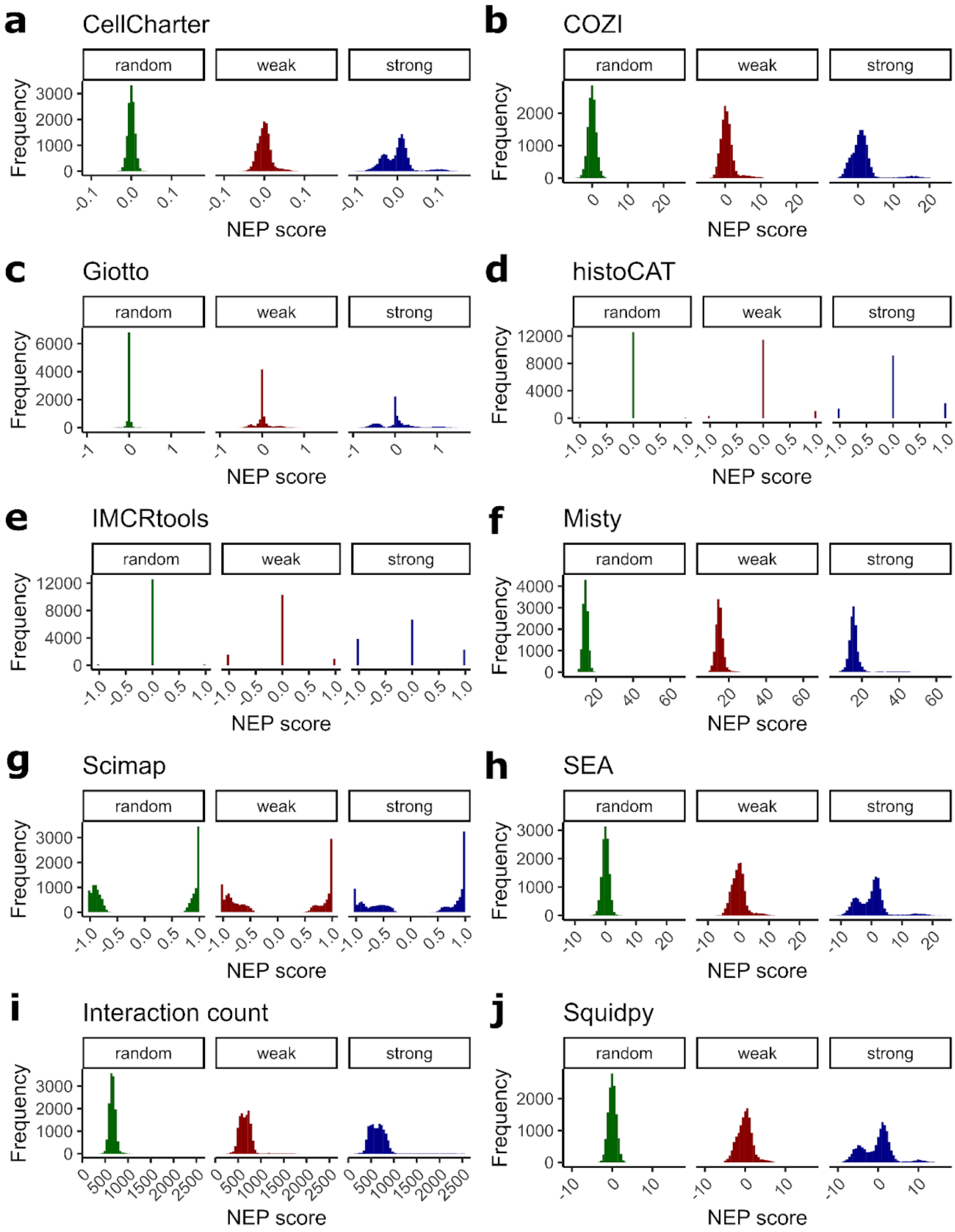

Supplementary Fig. 3: Overall NEP score frequencies across methods in simulated symmetric tissue cohorts. (a)-(j) Overall NEP score distributions per method across all cell type pairs in random (green), weak (red) and strong (blue) simulated tissue cohorts. Scores across all abundance groups are depicted. n = 100 samples per cohort were simulated and analyzed. Source data are provided as a Source Data file.

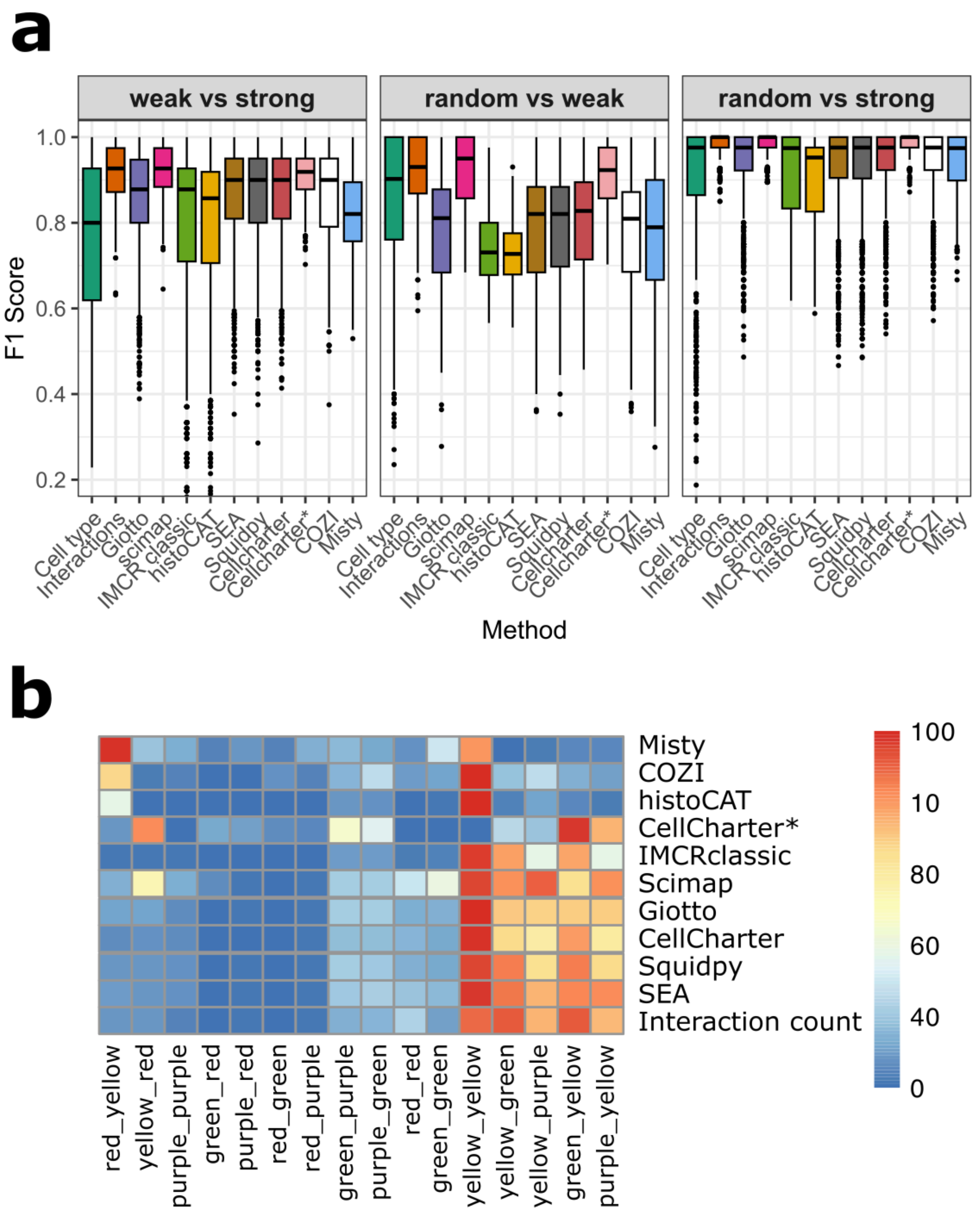

Supplementary Fig. 4: Overall cohort classification of the cross-preference dataset. (a) Mean F1-scores for cohort distinction tasks in the cross-preference dataset (red → yellow cell type), shown as boxplots across yellow cell abundance groups. Cohort comparisons include *weak vs. strong*, *random vs. weak*, and *random vs. strong*. Data are presented as box plots showing the median (centre line), interquartile range (box; 25th–75th percentiles), and whiskers extending to 1.5 × IQR. Outliers are shown as individual points. **(b)** Feature importances for distinguishing between random and strong cross-preference cohorts for all compared methods at 25% red and yellow cell type abundances. n = 100 samples

per cohort were simulated and analyzed. Color legend indicates the feature importance. Source data are provided as a Source Data file.

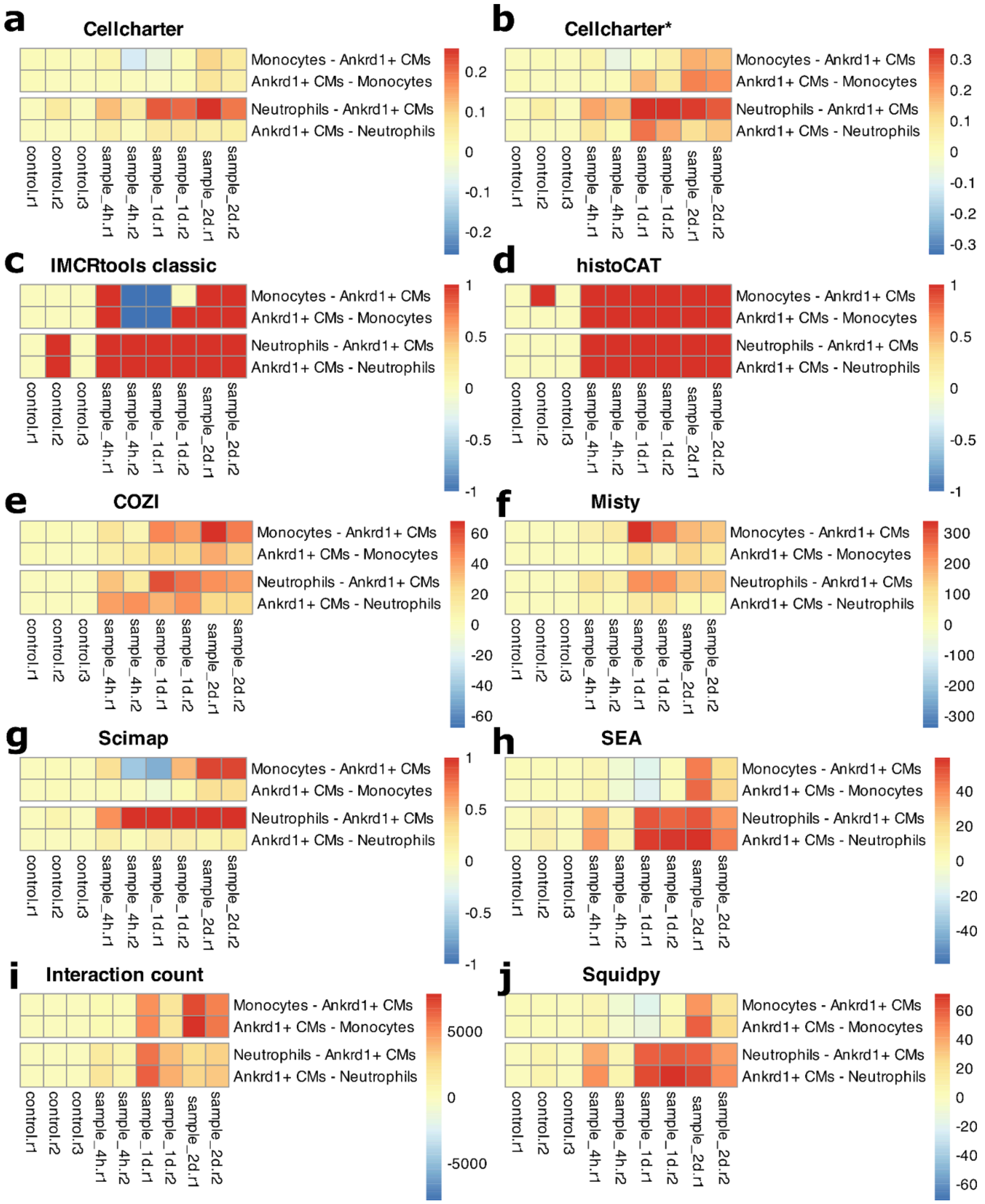

Supplementary Fig. 5: NEP scores of different methods for monocyte and neutrophil infiltration into the infarct region. NEP scores for monocytes and neutrophils with Ankrd1+ cells (stressed CMs) in control (n=3), 4h (n=2), 24h (n=2) and 48h (n=2) samples. Results are shown for **(a)-(h)** CellCharter, Cellcharter*, IMCRtools classic, HistoCAT, COZI, Misty, Scimap, SEA, Interaction count, and Squidpy. Color legends indicate the method specific NEP score. Source data are provided as a Source Data file.

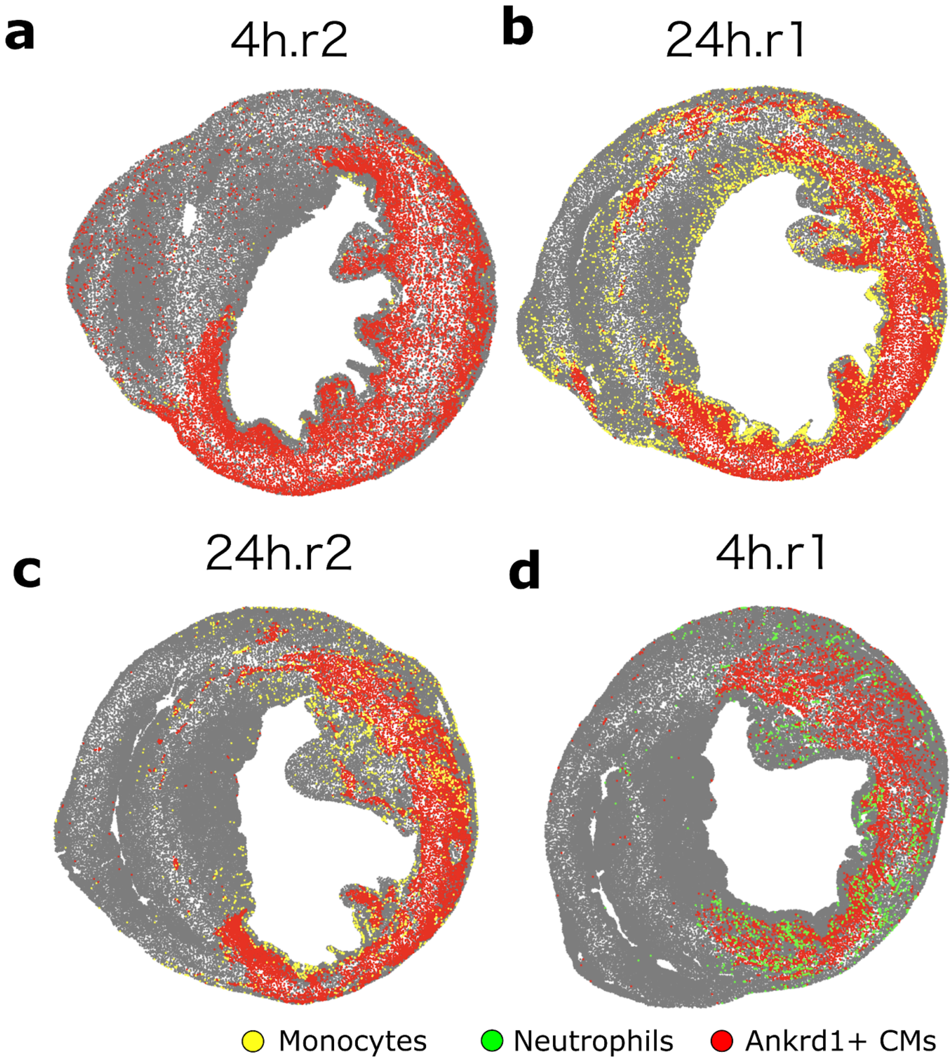

Supplementary Fig. 6: Mouse hearts at 4h and 24h after infarction to study monocyte and neutrophil infiltration. Samples at 4h for monocyte **(a)** and neutrophil **(d)** infiltration, Both samples at 24h for monocyte infiltration **(b, c)**. Monocytes (yellow), neutrophils (green) and Ankrd1+ cardiomyocytes (red) as scatter plots.

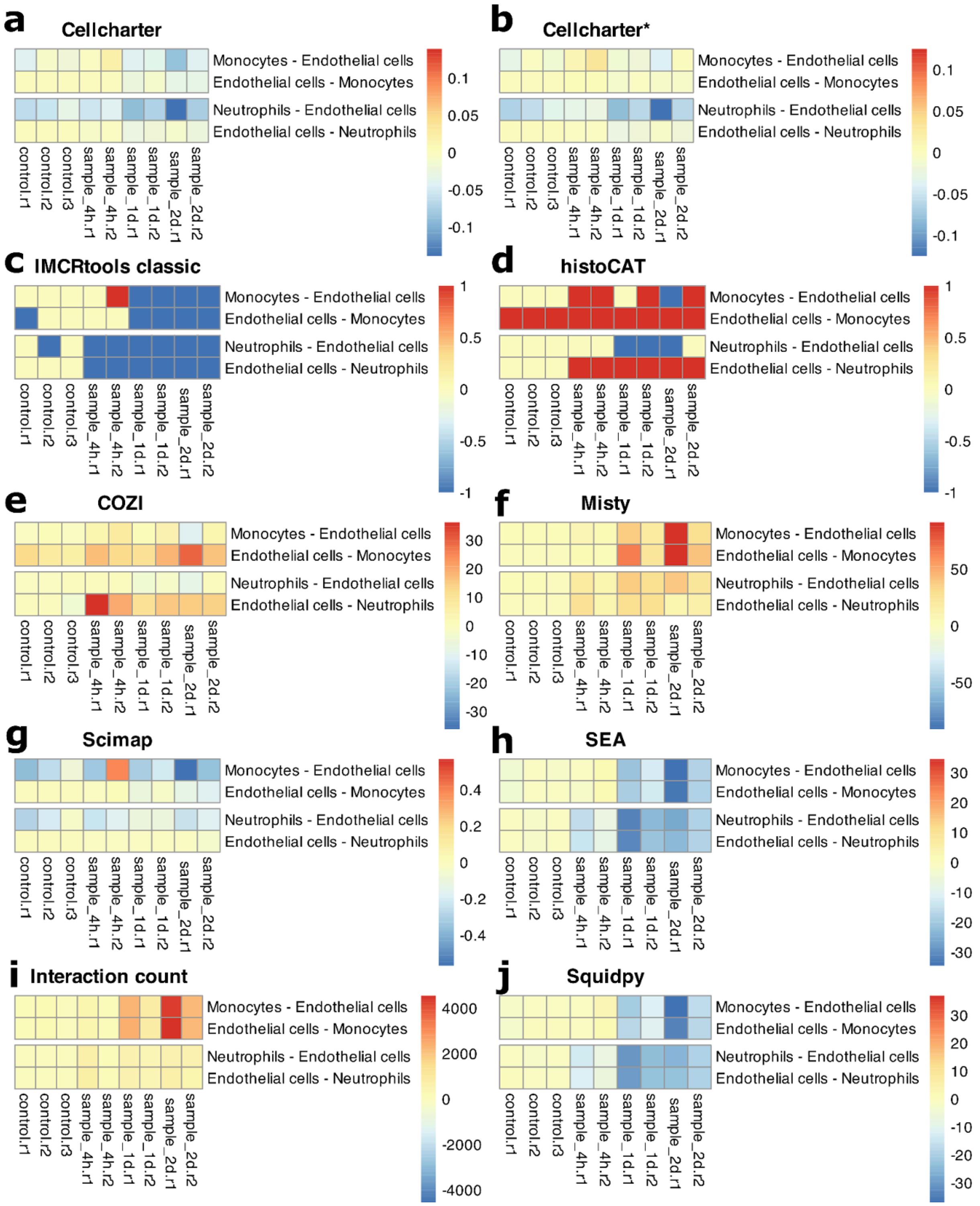

Supplementary Fig. 7: NEP scores of compared methods between monocytes and neutrophils with endothelial cells. NEP scores for monocytes and neutrophils with Endothelial cells in control (n=2), 4h (n=2), 24h (n=2) and 48h (n=2) samples. Results are shown for **(a)-(h)** CellCharter, Cellcharter*, IMCRtools classic, HistoCAT, COZI, Misty, Scimap, SEA, Interaction count, and Squidpy. Color legends indicate the method specific NEP score. Source data are provided as a Source Data file.

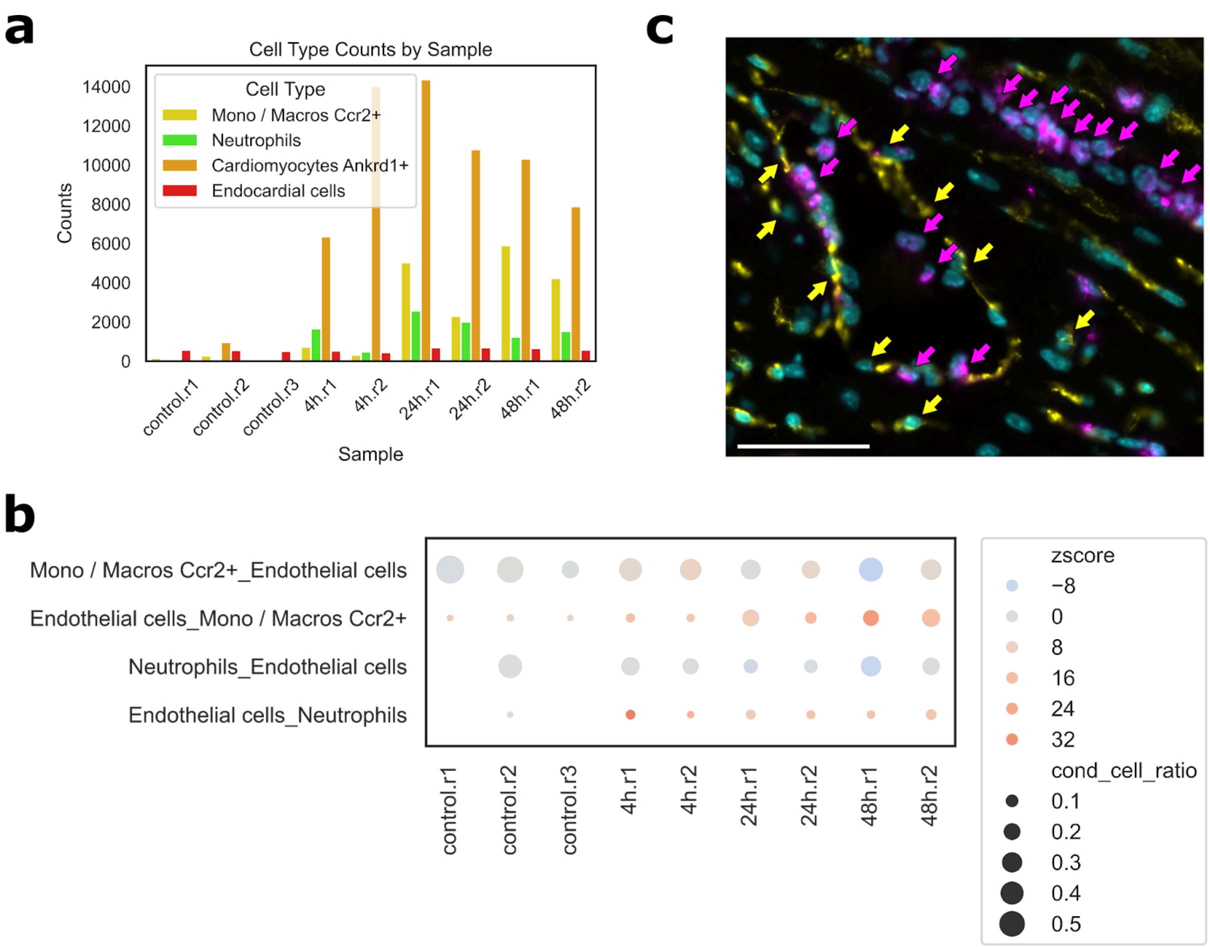

Supplementary Fig. 8: Neutrophil infiltration through the endocard. (a) Cell type counts across myocardial infarction (MI) dataset samples for monocytes, neutrophils, Ankrd1+ cardiomyocytes and endocardial cells. Control (n=3), 4h (n=2), 24h (n=2), and 48h (n=2) after infarction samples.**(b)** COZI neutrophil and monocyte NEP and CCR scores with endothelial cells for control, 4h, 24h, and 48h samples. **(c)** Neutrophil infiltration through blood vessels. DAPI (blue), MPO (magenta) and CD31 (yellow) mark cell nuclei, neutrophils and endothelial cells, respectively. The scale bar indicates 50 microns. Source data are provided as a Source Data file.

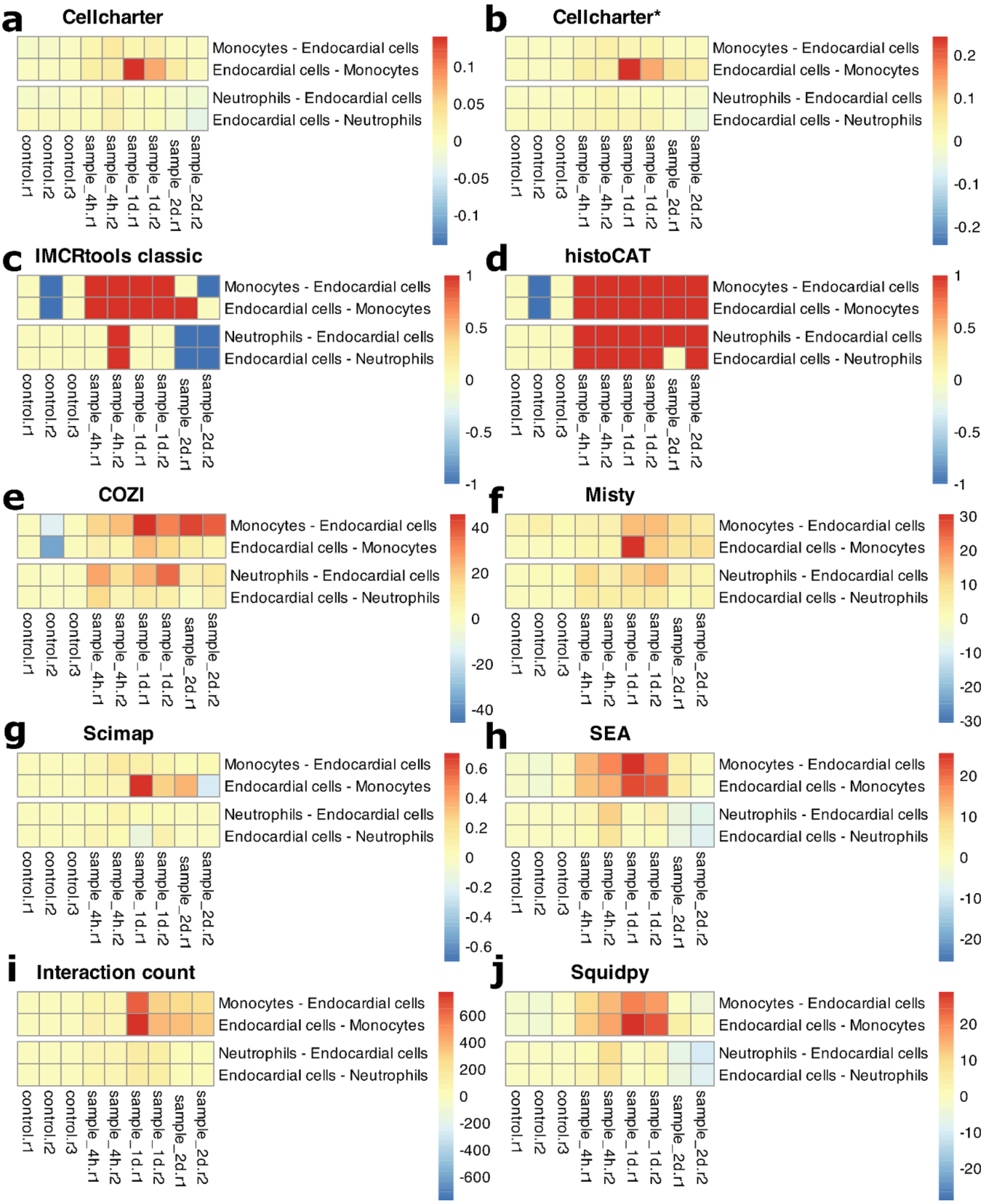

Supplementary Fig. 9: NEP scores of compared methods between monocytes and neutrophils with endocardial cells. NEP scores for monocytes and neutrophils with Endothelial cells in control (n=3), 4h (n=2), 24h (n=2) and 48h (n=2) samples. Scores are shown for **(a)-(h)** CellCharter, Cellcharter*, IMCRtools classic, HistoCAT, COZI, Misty, Scimap, SEA, Interaction count, and Squidpy. Color legends indicate the method specific NEP score. Source data are provided as a Source Data file.

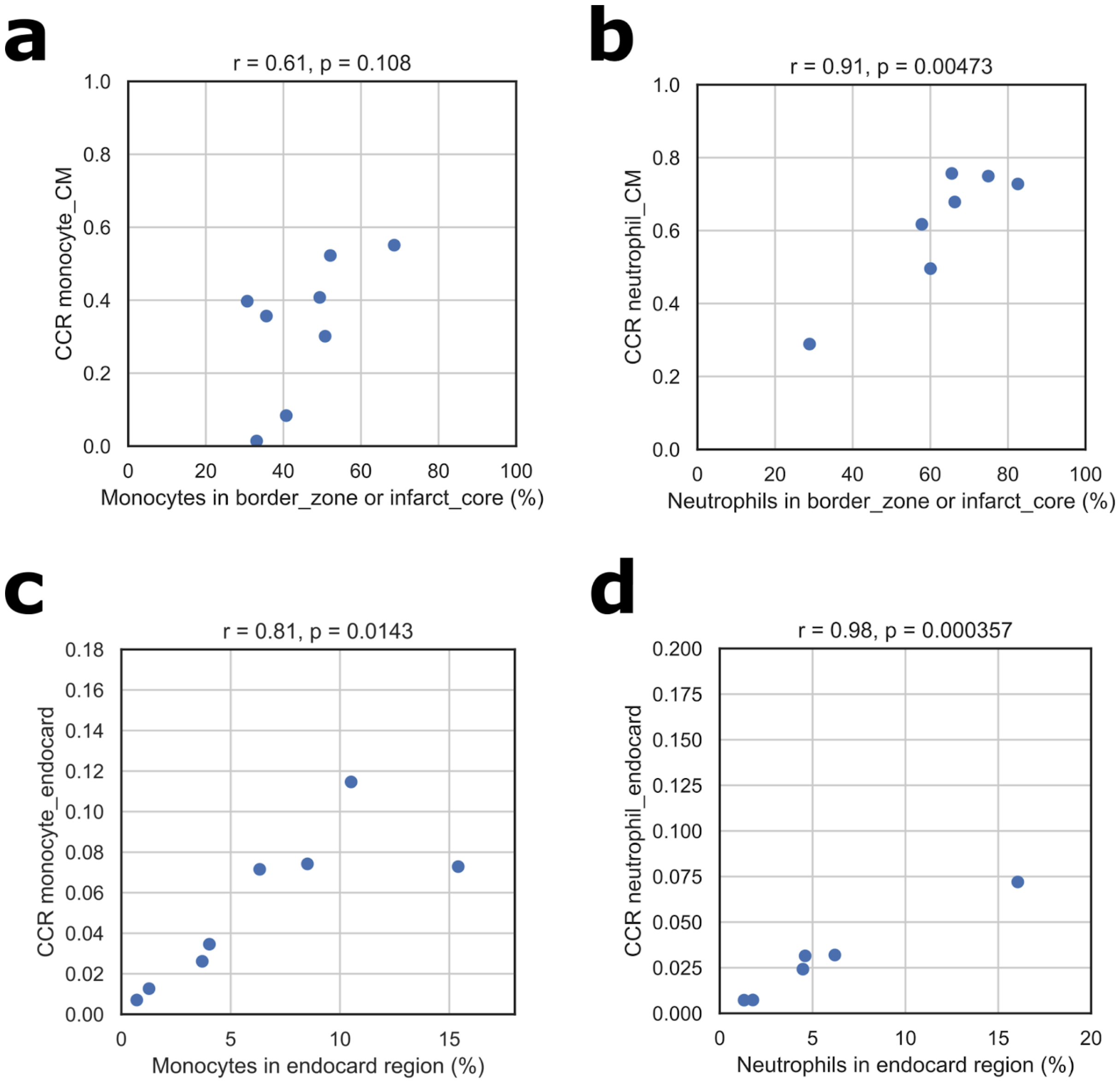

Supplementary Fig. 10: Correlations of CCR with annotated regions in myocardial infarction (MI) dataset. Pearson correlation between conditional cell ratio and percentage of the specific cell type in the manually annotated region. Control (n=3), 4h (n=2), 24h (n=2) and 48h (n=2) after infarction samples analysed. Only data points are displayed if the respective cell type was found in the indicated region. Correlations between **(a)** CCR of monocytes with stressed cardiomyocytes (CM) and the percentage of monocytes in border zone and infarct core regions **(b)** CCR of neutrophil with stressed CM and the percentage of neutrophils in border zone and infarct core regions, **(c)** CCR of monocytes with endocardial cells and the percentage of monocytes in the endocardial infarct zone **(d)** CCR of neutrophils with endocardial cells and the percentage of neutrophils in the endocardial infarct zone. Source data are provided as a Source Data file.

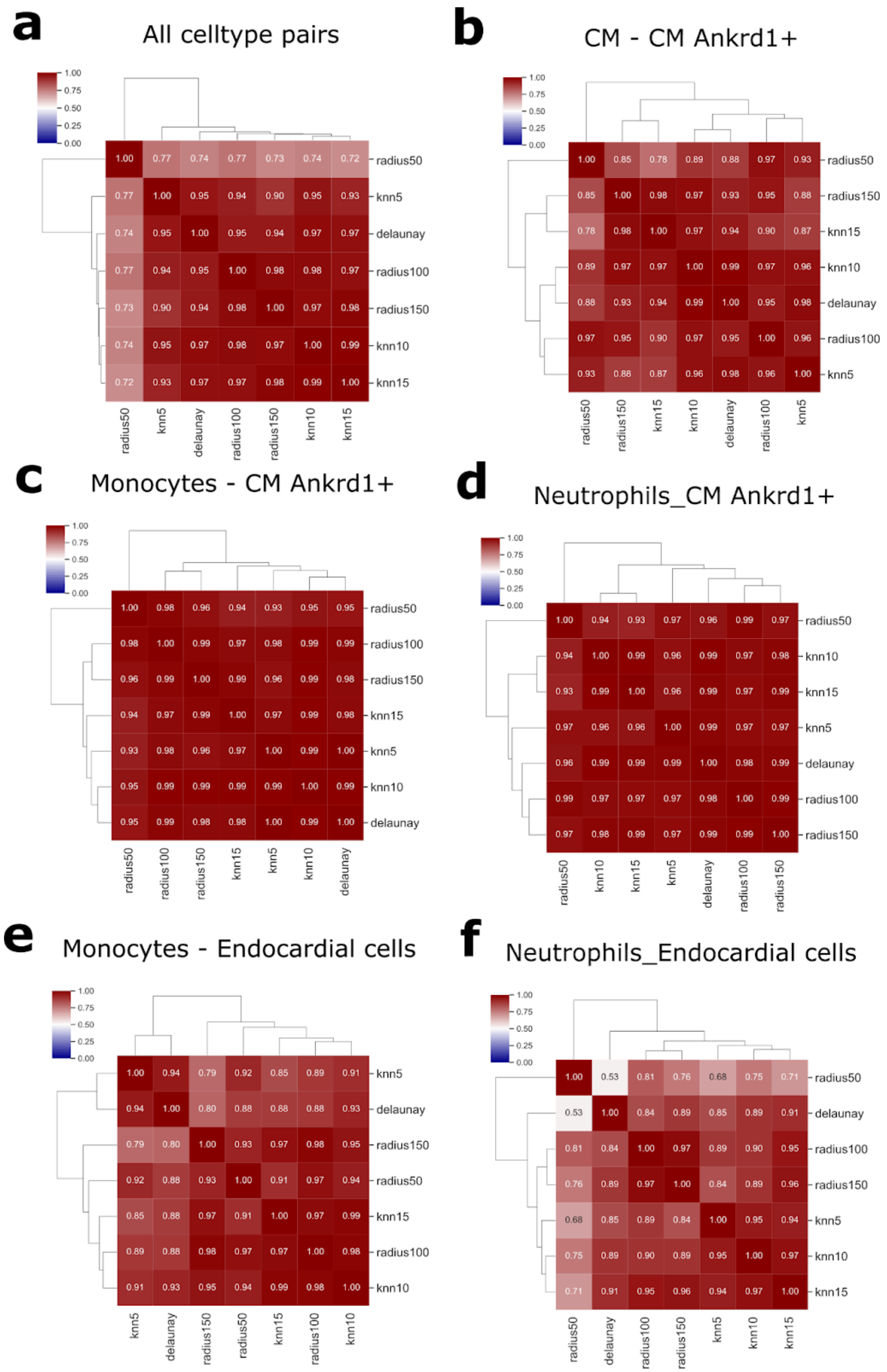
a
All celltype pairs
1.00
0.75
0.50
0.25
0.00
radius50
knn5
delaunay
radius100
radius150
knn10
knn15
b
CM - CM Ankrd1+
radius50
radius150
knn15
knn10
delaunay
radius100
knn5
c
Monocytes - CM Ankrd1+
radius50
radius100
radius150
knn15
knn5
knn10
delaunay
d
Neutrophils_CM Ankrd1+
radius50
knn10
knn15
knn5
delaunay
radius100
radius150
e
Monocytes - Endocardial cells
knn5
delaunay
radius150
radius50
knn15
radius100
knn10
f
Neutrophils_Endocardial cells
radius50
delaunay
radius100
radius150
knn5
knn10
knn15

Supplementary Figure 11: Overall robustness of COZI z-scores across neighborhood definitions and across all samples. (a–f) Robustness of COZI z-scores across neighborhood definitions of n=9 samples across conditions in the myocardial infarction (MI) dataset. COZI was calculated with different neighborhood definitions (fixed radius: 50, 100, 150; Delaunay; kNN with k = 5, 10, 15). Pearson correlations of NEP scores (color bar) were calcualted by taking the NEP score vectors of the indicated cell-type pairs. Correlations are shown for **(a)** all cell type pairs across all samples, and for specific pairs: **(b)** monocytes–stressed CM, **(c)** monocytes–endocardial cells, **(d)** stressed CM– stressed CM, **(e)** neutrophils–stressed CM, **(f)** neutrophils–endocardial cells. Source data are provided as a Source Data file.

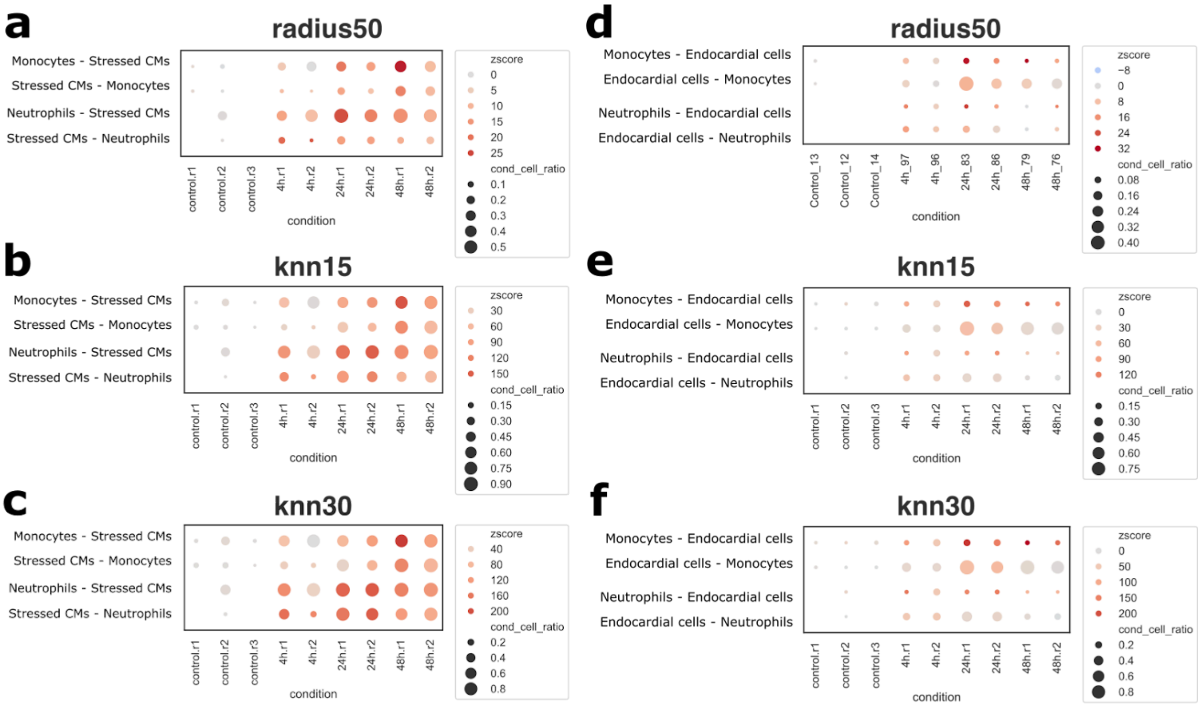

Supplementary Figure 12: COZI NEP findings across neighborhood scales on the MI dataset. COZI results on the MI dataset with varying neighborhood definitions to compare to Figure 6 c and f (kNN with k=5). COZI was calculated with different neighborhood definitions for small neighborhoods (fixed radius: 50) and larger neighborhoods (kNN with k = 15, 30). COZI z-scores (colorscale) with conditional cell ratio (dotsize) for the three neighborhood definitions for **(a-c)** monocyte and neutrophil NEP with stressed cardiomyocytes and **(d-f)** monocyte and neutrophil NEP with endocardial cells. NEP scores for control (n=3), 4h (n=2), 24h (n=2) and 48h (n=2) samples. Source data are provided as a Source Data file.
