# Supplementary material for: Comparison and optimization of cellular neighbor preference methods for quantitative tissue analysis
Source: Nat Commun. 2026 Apr 15;17:3514. doi: 10.1038/s41467-026-71699-z (PMC13083810; doi:10.1038/s41467-026-71699-z)
Supplement: Supplementary file 2 — Reporting Summary [file 41467_2026_71699_MOESM2_ESM.pdf]

nature portfolio

Corresponding author(s): Denis Schapiro

Last updated by author(s): 2026/03/10

Reporting Summary

Nature Portfolio wishes to improve the reproducibility of the work that we publish. This form provides structure for consistency and transparency in reporting. For further information on Nature Portfolio policies, see our Editorial Policies and the Editorial Policy Checklist.

Statistics

For all statistical analyses, confirm that the following items are present in the figure legend, table legend, main text, or Methods section.

n/a	Confirmed	
☐	☒	The exact sample size (*n*) for each experimental group/condition, given as a discrete number and unit of measurement
☐	☒	A statement on whether measurements were taken from distinct samples or whether the same sample was measured repeatedly
☐	☒	The statistical test(s) used AND whether they are one- or two-sided<br>*Only common tests should be described solely by name; describe more complex techniques in the Methods section.*
☒	☐	A description of all covariates tested
☐	☒	A description of any assumptions or corrections, such as tests of normality and adjustment for multiple comparisons
☐	☒	A full description of the statistical parameters including central tendency (e.g. means) or other basic estimates (e.g. regression coefficient) AND variation (e.g. standard deviation) or associated estimates of uncertainty (e.g. confidence intervals)
☐	☒	For null hypothesis testing, the test statistic (e.g. *F*, *t*, *r*) with confidence intervals, effect sizes, degrees of freedom and *P* value noted<br>*Give P values as exact values whenever suitable.*
☒	☐	For Bayesian analysis, information on the choice of priors and Markov chain Monte Carlo settings
☒	☐	For hierarchical and complex designs, identification of the appropriate level for tests and full reporting of outcomes
☐	☒	Estimates of effect sizes (e.g. Cohen's *d*, Pearson's *r*), indicating how they were calculated

Our web collection on statistics for biologists contains articles on many of the points above.

Software and code

Policy information about availability of computer code

Data collection	The in silico tissue (IST) and SpaSim simulated data used in this study are deposited in https://github.com/SchapiroLabor/NEP_comparison/simulated_data. The IST data used in this study can also be reproduced with the provided scripts in this repository: https://github.com/SchapiroLabor/NEP_IST_generation (https://doi.org/10.5281/zenodo.18887576). The SpaSim data used in this study can also be reproduced with the provided scripts in this repository: https://github.com/SchapiroLabor/NEP_SpaSim (https://doi.org/10.5281/zenodo.18887606).
Data analysis	We have compared the neighbor preference functions implemented in Giotto (v1.1.2) (https://github.com/SchapiroLabor/NEP_Giotto, https://doi.org/10.5281/zenodo.18887279), IMCRtools with classic and histoCAT (v1.8.0) (https://github.com/SchapiroLabor/NEP_IMCRtools, https://doi.org/10.5281/zenodo.18888850), MistyR (v1.10.0) (https://github.com/SchapiroLabor/NEP_MistyR, https://doi.org/10.5281/zenodo.18887408), CellCharter with and without homotypic interactions (v0.3.3) , Squidpy (v1.6.3) (both in https://github.com/SchapiroLabor/NEP_Squidpy, https://doi.org/10.5281/zenodo.18890966), Scimap (v2.1.3), SEA and COZI (https://github.com/SchapiroLabor/NEP_scimap, https://doi.org/10.5281/zenodo.18890905), both SEA and cozi implemented on a fork of scimap (v2.2.11) (https://github.com/SchapiroLabor/NEP_scimap) adapting the spatial_interaction() function (cozi v0.2.0).<br><br>We further made Cozi available as Python package (https://pypi.org/project/cozipy, https://doi.org/10.5281/zenodo.18887378) and R package (https://github.com/SchapiroLabor/coziR, https://doi.org/10.5281/zenodo.18925561).<br><br>We compared the output of the methods assessing their ability to distinguish different tissue cohorts using a random forest classifier in R (caret v6.0-94).<br><br>The overall code repository for comparing the methods used in this paper is available at https://github.com/SchapiroLabor/NEP_comparison, (https://doi.org/10.5281/zenodo.18889059) also containing seeds for reproducibility and all links to the NEP method specific repositories.

For manuscripts utilizing custom algorithms or software that are central to the research but not yet described in published literature, software must be made available to editors and reviewers. We strongly encourage code deposition in a community repository (e.g. GitHub). See the Nature Portfolio guidelines for submitting code & software for further information.

Data

Policy information about availability of data

All manuscripts must include a data availability statement. This statement should provide the following information, where applicable:

- Accession codes, unique identifiers, or web links for publicly available datasets
- A description of any restrictions on data availability
- For clinical datasets or third party data, please ensure that the statement adheres to our policy

The in silico tissue (IST) and SpaSim simulated data used in this study is deposited in https://github.com/SchapiroLabor/NEP_comparison/simulated_data. The provided IST data can also be reproduced with the provided scripts in this repository: https://github.com/SchapiroLabor/IST_generation_SCNA (https://doi.org/10.5281/zenodo.18887576). The SpaSim data used in this study can also be reproduced with the provided scripts in this repository: https://github.com/SchapiroLabor/NEP_SpaSim (https://doi.org/10.5281/zenodo.18887606).

The MI Sequential Immunofluorescence data used in this study is available via Synapse (project SynID : syn51449054): https://www.synapse.org/Synapse:syn51449054. The dataframe with phenotypes is available via Synapse: https://www.synapse.org/Synapse:syn65487454. The TNBC data used in this study can be found at https://www.angelolab.com/mibi-data. We used the processed data from https://github.com/psl-schaefer/report/tree/master/data. Source data are provided with the paper.

Research involving human participants, their data, or biological material

Policy information about studies with human participants or human data. See also policy information about sex, gender (identity/presentation), and sexual orientation and race, ethnicity and racism.

Reporting on sex and gender	Not applicable
Reporting on race, ethnicity, or other socially relevant groupings	Not applicable
Population characteristics	Not applicable
Recruitment	Not applicable
Ethics oversight	Not applicable

Note that full information on the approval of the study protocol must also be provided in the manuscript.

Field-specific reporting

Please select the one below that is the best fit for your research. If you are not sure, read the appropriate sections before making your selection.

☒ Life sciences ☐ Behavioural & social sciences ☐ Ecological, evolutionary & environmental sciences

For a reference copy of the document with all sections, see nature.com/documents/nr-reporting-summary-flat.pdf

Life sciences study design

All studies must disclose on these points even when the disclosure is negative.

Sample size	For cohort level analysis, we used two simulated IST datasets with 2400 samples each. We simulated 100 samples per set of tissue architecture parameters. We only simulated and used cell type annotations and not any marker intensity values.<br>For single sample analysis, we simulated three different datasets with SpaSim with varying levels of cell infiltration. Dataset I contained 7 images and datasets II and III contained 8 images each.<br><br>We used a mouse Lunaphore COMET dataset with 3 x control, 2 x 4h, 2 x 24h and 2 x 48h samples. For the analysis, we only used cell type annotations and no marker intensity values. We used a MIBI dataset with 40 samples. We only used cell type annotations and no marker intensity values.
Data exclusions	In the Lunaphore COMET dataset, we only excluded cell types labeled with "exclude" by the authors of the study.
Replication	To ensure reproducibility of our findings, we simulated 100 tissues per cohort in order to have enough data available. Second, we ran every analysis with the provided seeds at least twice to ensure identical results. In addition, we examined the effect of varying cell type abundances, cell-cell adjacencies and neighborhood definitions across methods in order to ensure robustness of the methods and their results.
Randomization	For the IST data, we ran all methods in a sample wise manner without the methods having any information about the cohort identity of the sample. In the random forest classification, we used 80% of the results as training and 20% as test data with five fold cross-validation. We trained and tested 100 times to ensure reproducibility. In addition, we generated a random baseline by randomly shuffling the cell type labels

	100 times to generate a random model baseline.
Blinding	Not applicable

Reporting for specific materials, systems and methods

We require information from authors about some types of materials, experimental systems and methods used in many studies. Here, indicate whether each material, system or method listed is relevant to your study. If you are not sure if a list item applies to your research, read the appropriate section before selecting a response.

Materials & experimental systems

n/a	Involved in the study
☒	☐ Antibodies
☒	☐ Eukaryotic cell lines
☒	☐ Palaeontology and archaeology
☒	☐ Animals and other organisms
☒	☐ Clinical data
☒	☐ Dual use research of concern
☒	☐ Plants

Methods

n/a	Involved in the study
☒	☐ ChIP-seq
☒	☐ Flow cytometry
☒	☐ MRI-based neuroimaging

Plants

Seed stocks	Not applicable
Novel plant genotypes	Not applicable
Authentication	Not applicable
